# Composition analysis (pick analysis) of waste generated from household: A pilot study in Ujjain city, India

**DOI:** 10.1016/j.heliyon.2023.e19902

**Published:** 2023-09-09

**Authors:** Madhanraj Kalyanasundaram, Kavya Krishnan, Surya Singh, Krushna Chandra Sahoo, Rachna Soni, Vivek Parashar, Namrata Mathankar, Ashish Pathak, Yogesh Sabde, Cecilia Stålsby Lundborg, Salla Atkins, Kamran Rousta, Vishal Diwan

**Affiliations:** a ICMR- National Institute of Epidemiology, Chennai, 600077, Tamil Nadu, India; b Division of Environmental Monitoring and Exposure Assessment (Water and Soil), ICMR – National Institute for Research in Environmental Health, Bhopal, 462030, Madhya Pradesh, India; c Health Technology Assessment in India Regional Hub, ICMR- Regional Medical Research Centre, Bhubaneswar, 751023, Odisha, India; d Ruxmaniben Deepchand Gardi Medical College, Ujjain, 456006, Madhya Pradesh, India; e Department of Global Public Health, Health Systems and Policy: Improving Use of Medicines, Karolinska Institutet, 171 77, Stockholm, Sweden; f Division of Environemtnal Health and Epidemiology, ICMR – National Institute for Research in Environmental Health, Bhopal, 462030, Madhya Pradesh, India; g Department of Global Public Health, Social Medicine Infectious Disease and Migration, Karolinska Institutet, 171 77, Stockholm, Sweden; h Global Health and Development, Health Sciences, Faculty of Social Sciences, Tampere University, 33100, Tampere, Finland; i Swedish Centre for Resource Recovery, University of Borås, 50332, Borås, Sweden

**Keywords:** Waste segregation, Slum and non-slum, Waste fraction, Pick analysis, Waste characterization

## Abstract

Waste segregation is an essential function in improving waste management. Waste segregation not only facilitates recycling and reduces waste going to landfills, rather it can benefit our environment and human in various ways. A pick analysis of waste composition is used to characterize the household waste stream and thus can analyze the segregation rate among the residents. In addition, it can measure the actual waste sorting behaviour at the household/community level. The objective of the study was to assess feasibility of a large-scale waste composition study, identify methodological and operational challenges, and estimate the resources needed to conduct the main waste composition study in order to obtain and get indicative figures about waste generation, composition, and miss-sorted proportions. The study team went door-to-door to collect waste in colour coded bags. We also collected the socio-demographic data of the households. The collected waste was weighed and segregated to analyze the waste composition. The analysis was done among 45 households, and it was found that the per capita waste generation per day is 0.25 kg (0.24 kg from slum and 0.27 kg from non-slum). Challenges identified in conducting waste composition study were lack of standard waste fraction classifications, difficulty in recruitment of personnel to conduct study due to social taboo around waste, challenge in co-coordinating with Ujjain Municipal Corporation waste collection vehicle for collection of waste. 53 household activities were completed in 5 and half hours with INR 24685 (USD 300.5). Pick analysis could be adopted by the Ujjain Municipal Corporation after cost effective analysis to generate precise estimate of waste generation, resource recovery, efficient resource allocation and will help in future interventions and informed policy decision making to improve segregation.

## Graphical abstract

**Figure:**
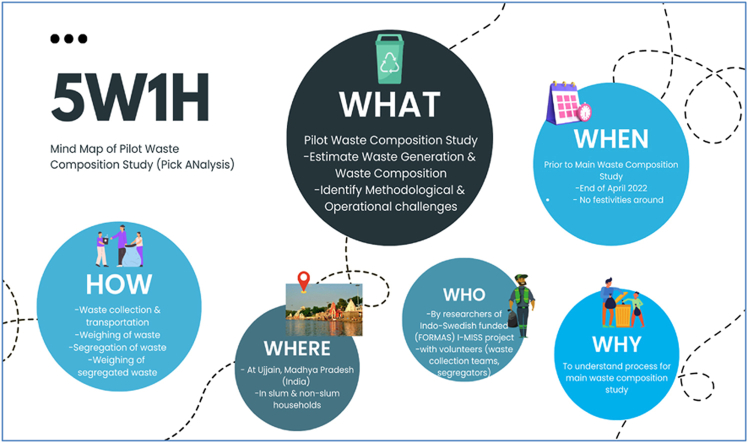

## 1 Introduction

Rapid urbanization and resultant massive waste generation, combined with lack of waste management resources has led to poor waste management systems in low- and -middle-income countries (LMICs) and has further exacerbated the issue of environmental degradation [1,2]. An estimated 29% (586 million tonnes per year) of global waste is generated from LMICs, whereas only 16% of that is treated through composting and recycling [3]. The remaining 84% undergoes unscientific and improper disposal such as burning, open dumping and landfilling; leading to emission of green house gases, global warming and climate change and ill effects on health and well-being of the planet and its beings [3].

India is the most populous country in the world, with increasing amounts of waste [4]. The Government of India (GoI) has mandated source segregation of waste, to attain sustainable waste management. Source segregation of waste is also one of the major criteria being used to rank Indian cities in *Swachh Survekshan*, the largest cleanliness survey being conducted by GoI as part of the Swachh Bharat Mission [5]. To effectively implement a source segregation strategy, understanding the composition of waste is crucial [6,7]. Waste composition determines the recycling and composting potential of the waste stream [8] and it varies from one region to another depending on diverse factors such as economic growth, culture, and climate [9].

Waste composition analysis or pick analysis is the technique of quantifying and categorizing different types of waste materials present in a waste stream [10,11]. There are several methods of waste composition or pick analysis which were outlined in various articles [[11], [12], [13]]. Some of the methods include the American Society for Testing and Materials (ASTM) method, Scott method by International Energy Agency, SWA-tool method by the European commission, method by the Swedish Association of Waste Management, and the quartering technique [12,14]. However, there is no standard international working method adopted for waste composition studies [12]. The steps that most studies followed include: Stratification, Sampling and Sorting [12]. Stratification divides population into homogenous subgroups. Sampling may be sampling from households, or from waste collection trucks [11,12]. The sorting categories are determined based on the adapted method and the waste type, its origin, disposal route and function [15]. Numerous studies have been conducted regarding the composition of waste on a global scale, with some focused on analyzing the proportions of food waste [16], hazardous waste [17], and other waste categories [10,16,[18], [19], [20]]. The composition of waste varies with the regions and changing seasons [9,21,22].

India generates an annual estimate of 62 million tons of waste, of which around half constitutes bio-degradable waste fraction [3,23]. Statistics of waste fractions at the aggregate level of India are made available by Central Pollution Control Board (CPCB) and also by the Ministry of Housing and Urban Affairs (MoHUA), GoI as a part of Swachh Bharat Mission. But waste management is the purview of Urban Local Bodies (ULB) and to meet out effective waste management strategy tailored to the needs of the urban area in question, understanding of waste generation patterns, composition of waste and waste management practices of households pertaining to that area or to that of a similar setting is a prerequisite. Using objective waste composition analysis at the local level can assist urban local bodies in planning and managing solid waste more efficiently. There is already some information about waste composition in India, for example a waste composition study conducted in Varanasi uncovered that a substantial proportion (32%) of the produced waste consisted of organic materials, followed by plastics and other recyclable matters [24], and an Uttarakhand study also reveals higher proportion of organic waste (57%) [25]. An assessment study done in the metro cities of India showed a higher proportion of compostable fraction (35–65%) than other waste fractions [18]. The change in the living standards due to economic development also influences the waste composition. Even though research shows a higher fraction of organic waste [26], there is also a trend of increasing rate of recyclable waste compared to previous years [18,27]. In addition, there is a lack of comprehensive studies that cover a wide range of regions in India. Investigating waste composition variations in different geographic areas, different seasons, and within different socio-economic groups could provide valuable insights for targeted waste management strategies.

Waste composition analysis at different times is essential to see the effectiveness of a behaviour change intervention with respect to waste segregation behaviour, waste minimization or use of waste as a resource and also to understand the recycling and composting potential of the generated waste. The present waste composition pilot study was designed as a part of a Swedish Research Council for Environment, Agricultural Sciences and Spatial Planning (FORMAS) funded project focusing on a community-based cluster randomized controlled trial to assess the effectiveness of improved information and volunteer support on segregation of solid waste at the household level in Ujjain City, Madhya Pradesh (I-MISS). The primary outcome of the I-MISS project is to compare the change in proportion of miss-sorted waste (waste that are sorted incorrectly) at different time points in the study when compared to baseline between the intervention and control groups [28]. This variable will be objectively assessed at group level across intervention and control groups by standard waste composition analysis (pick analysis). Hence, a pilot study was planned in Ujjain city with the following objectives.

• To assess the feasibility of pick analysis/waste composition study in Ujjain city
• To estimate the resources (manpower, materials and cost) required to conduct the main waste composition study in Ujjain city and
• To understand the operational difficulties in conducting the waste composition study

## 2 Methods

### 2.1 Study setting

Current pilot study's setting is the city of Ujjain, the fifth largest city of the state of Madhya Pradesh, India and having a population of 515,215 and over 102,401 households [29]. Ujjain holds a national rank of 10 among 382 Indian cities with population of more than 100,000 in *Swachh Survekshan* (Cleanliness survey) and has a 3-star rating (out of 7-stars) for Garbage Free Cities (GFC) ranking [5].

### 2.2 Waste generation and waste management system in Ujjain

The average solid waste generation in Ujjain city is 226 tons/day and 63% of total waste is from individual households [30]. Ujjain's waste management system entails daily door-to-door waste collection from each household using a four-compartment vehicle [31], this service is being provided by the local municipal body, Ujjain Municipal Corporation (UMC) to the residents of the city. The vehicle compartments are colour-coded based on the types of waste generated by households. Green and blue colour compartments located along the sides of the vehicle are for wet and dry waste collection, respectively. The vehicle also has small red and yellow sections at its back for hazardous and sanitary wastes, respectively [31]. UMC waste collection employees who accompany the vehicle instruct household members to dispose of their household waste in the appropriate compartment in the vehicle and thus engage in fostering the waste segregation behaviour of residents [32].

### 2.3 Study design

We conducted a community-based cross sectional study in the administrative boundary of Ujjain Municipal Corporation.

### 2.4 Study population

Households located within the administrative boundary of Ujjain Municipal Corporation was our study population. We included the households with at least one member above 18 years of age, located in the selected ward under Ujjain Municipal Corporation. Households whose waste mixes with waste generated from commercial use (shops, markets, salon, school, clinics etc.) and industrial use were excluded. We followed the definition of household given by the Census of India, i.e., A “household is usually a group of persons who normally live together and take their meals from a common kitchen unless the exigencies of work prevent any of them from doing so”. There may be one-member households, two-member households, or multi-member households [33].

## 2.5 Sample size and sampling strategy

As this was a pilot study, we did not calculate sample size. One ward (administrative unit created under UMC) was selected conveniently. A list of streets stratified by slum area and non-slum area of the selected ward was retrieved from the UMC authorities. One street in the slum area and one street in the non-slum area were selected by simple random sampling. All the households in the selected streets that fit our inclusion criteria were included for the survey. We followed the slum definition given by the Census of India [34,35]. According to that, slums are categorized and defined as one of the following three types.

1. **“Notified slums** (All notified areas in a town or city notified as ‘Slum’ by State, Union Territory (UT) Administration or Local Government under any Act including a ‘Slum Act')"
2. **“Recognized slums** (All areas recognized as ‘Slum’ by State, UT Administration or Local Government, Housing and Slum Boards-not been formally notified as slum under any act)"
3. **“Identified slums** (A compact area of at least 300 population or about 60–70 households of poorly built congested tenements, in an unhygienic environment, usually with inadequate infrastructure and lacking in proper sanitary and drinking water facilities-identified by the census of India)"

Any household located within the above-mentioned areas were considered a slum household.

## 3 Data collection methods

Data collection was done during the end of April 2022. We ensured that there were no special events or festivals during data collection to ensure that the routine waste generation rate of the households didn't get affected.

### 3.1 Preparatory activities

We obtained the necessary permissions from the authorities. Waste collection was conducted in coordination with the Ujjain Municipal Corporation (UMC). UMC waste collection vehicle was assigned for waste collection, and the driver and helper were informed about the study and the purpose of collecting waste separately from selected households. To ensure that the accumulated waste (generated on previous days) was not included in the waste composition analysis, wastes were taken from all study households a day prior to the pilot study and discarded.

### 3.2 Human **resource** and training

Field staff were recruited and trained on waste collection, transportation and segregation procedures. A brief questionnaire was developed to collect the socio-demographic details and waste segregation behaviour of the households which was administered by one of the researchers.

### 3.3 Steps of pick analysis (Fig. 1)

1. Waste collection

**Fig. 1:**
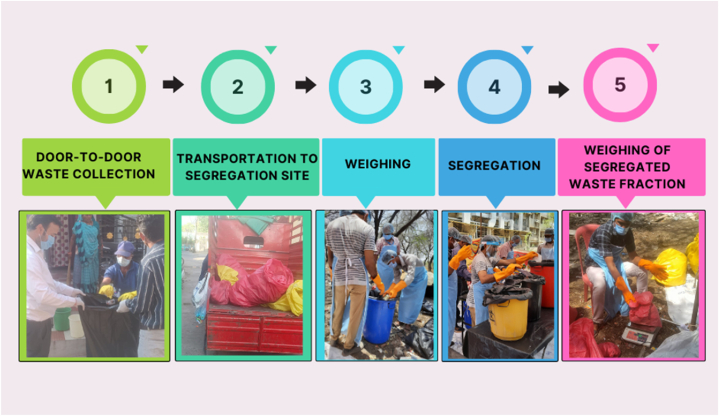
Steps of pilot pick analysis/waste composition study.

A team of five, supervised by the investigators collected the waste from the selected households at their doorstep. The waste was collected in colour-coded and House ID marked bags for wet, dry, mixed, hazardous and sanitary waste. All the waste handlers used appropriate personal protective equipment/kits like masks, boots, hand gloves and face shields/goggles.

One of the team members marked the waste category (wet/dry/sanitary, etc.) based on that reported by the household member who disposed of the waste on the day of data collection. The bags were locked immediately after the collection to prevent spillage and placed in the collection vehicle. Concurrently, one of the team members collected socio-demographic information such as total family members, total family income, education & occupation of the head of the household and waste segregation behaviour such as whether they follow segregation, availability of dustbins and if available, number of bins and primary person responsible for waste segregation.

2. Transportation:

A pick-up vehicle was arranged for transportation of the collected waste bags. The vehicle accompanied the waste collectors. Collected and locked waste bags were kept in the vehicle and were immediately transported to the segregation (waste composition analysis) site at R. D. Gardi Medical College, Ujjain.

3. Weighing at Segregation site:

An enclosure was temporarily erected at the segregation site to perform safe and systematic pick analysis. Tables/platforms and other related items were also arranged for segregation. Dustbins were used and placed on platforms after proper marking based on the waste fractions' classification. Surface mount electronic weighing machine, earlier checked for accuracy and calibrated, was used.

First, each collected dry waste bag (colour coded and marked with house ID) was weighed and the same was documented in the proforma. Once, household-level documentation was over, all the individual household-level dry wastes were mixed in a big dustbin and was used for waste composition/sorting analysis. The weights of the empty dustbin with the poly bag were measured and weight of the dustbin with waste was measured. The difference between the two gave the actual weight of the wastes. The similar process was repeated for the other categories of waste as well. Analysis was done separately for slum and non-slum households (House id marked on the waste bag enabled us to do the same). Three individuals from the research team involved in the weighing process (one documented the weights and other details and other two involved in weighing and emptying the waste in big dustbins (for waste composition analysis).

4. Segregation

*In waste fractions:* 5 trained personnel with protective gears such as aprons, masks, face shields, gloves and boots carried out the segregation/sorting/waste composition analysis process. Waste was categorized into different fractions as given in Table 1.

**Table 1:** Major waste categories and its fractions.

Major Waste Categories	Waste Categories Operational Definitions	Further Waste Fractions (N = 11)
1. Wet waste	Biodegradable waste that can be composted	i. Kitchen waste/food waste
		ii. Garden waste
2. Dry waste	Non-biodegradable waste, often recyclable	iii. Plastics & plastic packaging
		iv. Paper & cardboard
		v. Metal
		vi. Glass
		vii. Textiles
		viii. Other dry waste
3. Sanitary waste	Things that are soiled with biological fluids	ix. Sanitary waste
4. Hazardous waste	Wastes that if mixed can either create hazardous compounds or can harm the waste handlers	x. Chemical wastes (paint, oils, pesticides, pharmaceuticals/medicine,repellent etc.)
		xi. Sharp objects (blades, razors, insulin syringes, etc)

Eleven marked bins were placed on the platforms. The operational definition of each fraction was clearly explained to the staff. Each lot of waste (a big dustbin with waste) was emptied (after weighing) and spread evenly on the platform, and the trained personnel segregated the waste into eleven waste fraction bins. Then, each waste fraction was weighed and marked on the proforma. A similar process was repeated for all the lots (wet, dry, sanitary, hazardous, and mixed).

## 4 Outcome

We assessed the resources required in terms of human resources and logistics required to complete the pick analysis of the selected number of households. The feasibility indicators are a) proportion of the surveyed households that provided waste to the team for waste composition analysis, b) the ability to compute waste generation per capita per day, describe the waste fraction, and compute miss-sorted percentage. We identified the operational challenges and the methodological challenges to be addressed for the main pick analysis study. In addition, we estimated the waste generation rate per household, type of waste fraction, and miss-sorted waste proportion among the surveyed households.

### 4.1 Data entry and analysis

The data was entered in MS Excel and analyzed. Per capita and per household waste generation (total/dry/wet waste), proportion of each fraction of the waste was calculated in total and disaggregated by the slum and non-slum households as well. Proportion of the miss-sorted waste in dry and wet waste bins was calculated.

## 5 Results

Out of 53 households surveyed, waste was collected from 45 households. There was no waste generated in eight households (5 slum and 3 non-slum) on the day of survey. Waste composition analysis was done with waste collected from 15 slum household and 30 non-slum households.

The required resources and time taken for each core activity of this pilot pick analysis are detailed in Table 2. It took about five and a half hours to complete the activity of pick analysis in 53 households, with a total of 9 members in the research team including 5 ad-hoc staff hired for the activity with a total cost coming up to INR 24685 (USD 300.5).

**Table 2:** Core activities and resources required for pilot pick analysis.

Sl. No	Core activities	Time taken in minutes	Human resources	Materials required	Total Cost	
					Human Resource INR (USD)	Materials INR (USD)
1	Waste collection:	120	5 (Field staff)^a^	Large waste bin bags, rubber bands, gloves, face mask	3000 (36.5)	2785 (33.9)
2	Transportation to segregation site:	30	1 (Driver)	Vehicle with storage space	–	8120 (98.94)
3	Weighing of waste	30	3 (Field staff)^a^	Weighing machine	–	3200 (38.99)
4	Segregation of waste into fractions & their weighing:	150	5 (Field staff)^a^	Protective Gears: Gloves, Face shield, Boots, Apron, Head cover; Dustbins, Land space (segregation site)^#^	1800 (21.9)	5780 (70.47)
	Total	330	8 personnel^a^	–	4800 (58.4)	19885 (242.1)
					Total cost: 24685 (300.5)	

a Each may be involved in multiple core activities. To complete all the core activities, eight personnel are required. # Land provided by the ULB, so cost for the land not included.

**Fig. 2:**
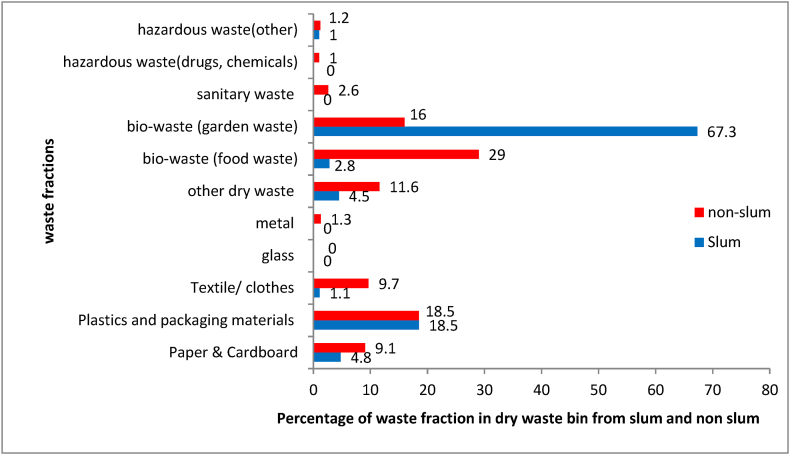
Comparison of generated waste fractions in dry waste bin between the slum (N = 15) and non-slum households (N = 30).

We were able to estimate the daily waste generation rate per household, the per capita per day waste generation rate (Table 3) and the proportion of miss-sorted waste in dry and wet bin. Total waste generated was 66.5 kg from 45 households per day. The total per capita per day waste generation was found to be 0.25 kg and total waste generation per household per day was 1.55 kg, of which 0.97 kg was wet waste and 0.58 kg was dry waste (self-reported as wet & dry). The households did not report any sanitary and hazardous labelled wastes during the survey. The findings from this pilot study indicate that a significant portion of the dry waste collected is mixed with wet waste, with bio waste (garden waste (32%) and food waste (20%) being the major components. Plastics and plastic packaging, papers and cardboards, textile/clothes are also present, though in relatively smaller proportions. The comparison of waste fractions in dry waste bin between slum and non-slum households (Fig. 2) shows that food waste proportion is lower in slum households, while garden waste proportion is higher in slum households.

**Table 3:** Waste generation rate among surveyed households.

Sl. No	Waste generation	All households (N = 45)	Slum households (N = 15)	Non-slum households (N = 30)
1	Total waste generation per household per day (in kg)	1.55	1.37	1.64
2	Dry waste generation per household per day (in kg)	0.58	0.50	0.62
3	Wet waste generation per household per day (in kg)	0.97	0.87	1.03
4	Total waste generation per capita per day (in kg)	0.25	0.24	0.27
5	Dry waste generation per capita per day (in kg)	0.09	0.09	0.10
6	Wet waste generation per capita per day (in kg)	0.16	0.15	0.17

**Fig. 3:**
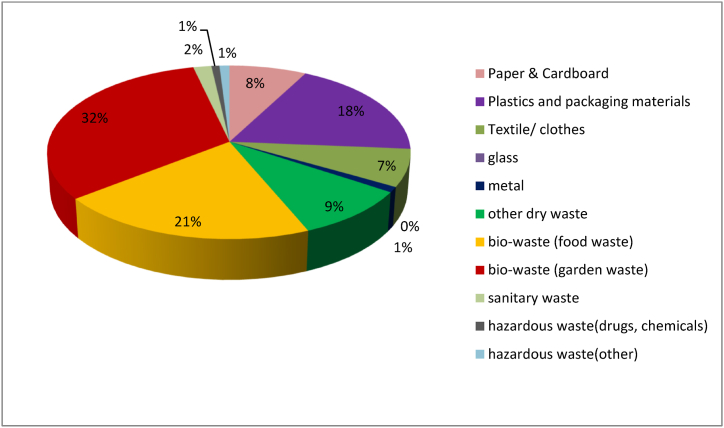
Proportion of waste fraction(s) generated among the study households (N = 45).

The mis-sorted percentage in dry waste bins was 56.4% overall and 49.7% and 71.1% among the non-slum and slum households respectively. Miss-sorted percentage was very low in wet waste bin, 3.6% overall and 7.4% & 1.7% in slum and non-slum households respectively. The proportion of each waste fraction in dry waste bin generated from households is shown in Fig. 3.

Based on the data obtained from administered questionnaire it was found that the majority of the households were aware about the waste segregation into dry and wet waste categories. The use of four bin systems was not followed by the community. In slum households, although they were aware about the dry and wet waste category, the understanding about the classification was not adequate. Most of the slum households had only one dustbin in which they tried to segregate dry and wet waste kept in different polythene bags. In slum households, few people dumped waste in open areas or empty plots or drains for their convenience, one of the reasons being that timing of the waste collection vehicle was not conducive for them as they go to work during that time. Many households, as part of their daily routine, gave kitchen waste to stray cattle or other stray animals like dogs. Few of the enablers and barriers to conduct waste composition study in Ujjain city are listed in Table 4.

**Table 4:** Enablers and challenges for conducting waste composition analysis.

Enablers	Barriers/challenges
Support from the Urban Local Bodies (ULBs) Door to door waste collection (daily) by the ULBs Availability of route plan by the ULB waste collection vehicle Apparently acceptable level of awareness about waste category and segregation in the study population	Operational challenges (manpower) Difficulty in recruiting field team (due to cultural taboo around waste collection) Training of waste collection and waste segregation team Operational challenges (materials) Co-ordination with the municipality waste collection vehicle Waste collection bag size/lock to prevent spillage Methodological challenges In the study setting, lack of standard waste classification system including comprehensive list of items used routinely by the households In the study setting, lack of standard colour coding system for waste fractions

Understanding of waste types, its classification, and colour-coding system is utmost important in analyzing waste fractions. Currently in India, there is no standard waste classification system or standard colour-coding system for dustbins which became one of the significant challenges of the study. Lack of public awareness on waste and its proper management and social taboo around waste collection made it difficult to recruit the ad-hoc personnel for the study for performing waste collection and segregation procedures. Co-ordination with the local municipal corporation and the waste collection vehicle was important. The regular route plan of the waste collection vehicle was studied, and the door to door waste collection by the research team was coordinated with them as per their schedule of visiting the selected households (of the study). The co-ordination was challenging so as to cover all the households such that they gave their waste to the research team and not the waste collection vehicle. The size of the bin bags taken for waste collection was small compared to the mouth of the dustbins of the households and was leading to the spillage of the waste during waste collection process. Also, there was no arrangement done for locking of the bin bags with collected waste on the day when the waste was picked and discarded. This was rectified on the pilot pick analysis day by getting larger bin bags and getting rubber bands for locking the bags.

## 6 Discussion

We conducted this pilot study to demonstrate feasibility, gain experience, identify methodological and operational challenges, and estimate the resources needed to conduct the main waste composition study in 880 households in Ujjain (as part of the I-MISS cluster-RCT) [28].

This study demonstrated the feasibility of this method by analyzing the composition of the waste generated by 45 households in Ujjain, estimating the household waste generation rate, describing the different fractions of the waste generated, and estimating the percentage of miss-sorted waste among households surveyed. We did not encounter any refusals from the household to provide their waste for composting analysis. Eight of the 53 households surveyed did not provide waste since sufficient waste was not accumulated on the day of the survey. The pick analysis of 53 households took 5 and a half hours, involving 9 team members, including 5 ad-hoc staff, with a total cost of INR 24685 (USD 300.5). On the basis of these data, an appropriate budgeting of resources for the main study (where we plan to study 880 households) needs to be planned. A cost-benefit analysis of this activity is necessary to determine whether the local municipality should conduct this activity periodically to objectively assess and characterize waste generated in the community.

Data generated from this study are indicative and cannot be generalized since the sample size is very small and not representative. Based on the indicative figures, it appears that non-slum households generated more waste than slum households. We have not compared these findings to other published literature as they are just indicative. Estimating these variables successfully is one indication of feasibility of pick analysis.

Regarding challenges identified, we experienced greater difficulty to recruit personnel to work in this sector, especially to do manual jobs of waste collection and segregation due to the prevailing cultural taboo associated with the activity of waste collection. Literature supports the existence of this kind of taboo in most of the Indian societies [26]. Creating a waste fraction classification for training and operationalization of segregation was a challenge as there are no concrete guidelines available in India that can enlist all waste items and their respective categories of waste. Central Pollution Control Board (CPCB) of India has published guidelines for management of various wastes as per Solid Waste Management Rules, 2016 (which can be found here - https://cpcb.nic.in/waste-management/[36]) which gives an idea about the operational definitions of the different types of wastes and few examples of such wastes, but it does not contain an exhaustive list of items or items that are routinely used by the households and confusing to classify (for ex: soap, thermocol, bangles, etc.). There is a need to update these lists and make them more exhaustive or at least consider to include such items that may be confusing to classify into any particular category of waste. Also, various ULBs follow different number of bin systems and different colour coding system which also poses a challenge for conducting and interpreting such waste composition studies. As per Swachh Bharat manual, green bin is recommended for bio-degradable waste, white bin for dry waste, and the hazardous waste will be depending on respective ULBs [36,37]. But many people depend on news articles for information and some of the news articles suggest the colours green (biodegradable), blue (recyclable) and yellow (other waste) [[38], [39], [40]]. Similarly, in some others, it is green (biodegradable waste), black (e-waste) and blue (plastic and metal waste) [38]. These types of information may mislead the community. The waste classification framework should be standardized along with giving appropriate examples so that it could be a guide for all the ULBs**.**

In an earlier study conducted in Ujjain, it was found that residents resort to improper waste disposal such as dumping in open places or in the drains due to variety of reasons and some of the wet waste is also fed to the stray cattle and other animals such as dogs [31], due to this it can become difficult to estimate the waste generation rate. This should be accounted during quantification of waste generation in the main study. Additionally, general instructions can be given to households to avoid such activities during the waste composition exercise as one of the preparatory activities.

The support from the provider side (Ujjain Municipal Corporation) was an enabler. Ujjain Municipal Corporation provides service of door to door waste collection to the citizens of the Ujjain city for a service fees. The routes and the route plans of the relevant waste collection vehicles were thus studied thoroughly to ensure that the research team accompanied the vehicle to the selected households, thus it did not create inconvenience for the selected residents and it also ensured that the research team got the waste from those households and they did not dispose it off in the UMC vehicle.

### 6.1 Lesson learnt from the pilot study for the main study

Recruitment of the personnel to carry out waste collection and segregation may take time, all resources can be used to recruit them and enough time to be reserved for the process of recruitment for the main study. Training of the personnel to be conducted by showing each and every sample of waste that can be expected from households and classification category to be ascertained as per the classification system followed by the study. Households should be given clear instructions to not give the waste to waste collection vehicle, to be fed to cattle/animals, or to be disposed of by any other means but to be given to the research team. Co-ordination with UMC waste collection vehicle to be done in order to ensure that waste collection of all the selected households can be done conveniently for the 7-day long main study. Bin bags to be carried for household waste collection should be large enough to hold the mouth of the dustbins of the households. The locking system for bin bags should be proper to ensure that the collected waste does not spill.

### 6.2 Strength and Limitations

The present study included both slum and non-slum populations to compare and analyze the daily waste generation and to understand the feasibility of conducting large scale study in both areas. This pilot is first of its kind waste composition study to be conducted scientifically in tier-2 city of India giving an insight into waste management and segregation behaviour of people.

As this study was a pilot study, we have used one ward for convenience. So the results and interpretation with a small sample size may not be generalized.

## 7 Conclusion

In this study, waste composition study (pick analysis) was carried out in the Ujjain city of Madhya Pradesh (India). Per capita waste generation per day was found to be 0.25 kg. Further, miss-sorted proportion was high in dry waste bins while low in the wet waste bins. The use of 4 bin system was not seen in the community. To conclude, it is feasible to collect wastes and conduct pick/waste composition analysis of waste generated from 50 households in a day. Waste composition study are feasible in terms of resources required and operationalization, thus can be taken up as periodic exercises by the urban local bodies in order to obtain improved estimates of waste & waste fractions generation, and understand public's waste management behaviour. The results of such exercises can then be utilised to implement resource recovery and circular economy operations, efficient resource allocation, planning and management of Solid Waste Management activities and can help in informed policy decisions and policy changes.

## Ethical approval

The study was approved by Institutional Ethical Committee of ICMR – National Institute for Research in Environmental Health, Bhopal (NIREH/BPL/IEC/2020–21/41 dated April 21, 2020) and Institutional ethics committee of R D Gardi Medical College, Ujjain (03/2020 dated March 12, 2020). The study is also approved by ICMR-HMSC (2020–9308-). Participants were informed about the study and written consent was taken prior to data collection.

## Funding

The work was supported by 10.13039/501100004359Swedish Research Council (FORMAS) for Environment, Agricultural Sciences, and Spatial Planning (grant no: 2019–00439). The funders had no role in study design, data collection and analysis, decision to publish, or preparation of the manuscript.

## Author contribution statement

**Madhanraj Kalyanasundaram; Vishal Diwan**: Conceived and designed the experiments; Performed the experiments; Analyzed and interpreted the data; Contributed reagents, materials, analysis tools or data; Wrote the paper. **Kavya Krishnan**: Conceived and designed the experiments; Performed the experiments; Analyzed and interpreted the data; Wrote the paper. **Surya Singh**: Conceived and designed the experiments; Performed the experiments; Analyzed and interpreted the data; Contributed reagents, materials, analysis tools or data. **Krushna Chandra Sahoo**: **Conceived and designed the experiments**; Contributed reagents, materials, analysis tools or data. **Rachna Soni**: Conceived and designed the experiments; Analyzed and interpreted the data; Wrote the paper. **Vivek Parashar; Ashish Pathak**: Conceived and designed the experiments; Performed the experiments; Contributed reagents, materials, analysis tools or data. **Namrata Mathankar**: Performed the experiments. **Yogesh Sabde**: Conceived and designed the experiments; Analyzed and interpreted the data; Contributed reagents, materials, analysis tools or data. **Kamran Rousta; Salla Atkins; Cecilia Stalsby Lundborg**: Conceived and designed the experiments, Contributed reagents, materials, analysis tools or data.

## Data availability statement

Data will be made available on request.

## Declaration of competing interest

The authors declare that they have no known competing financial interests or personal relationships that could have appeared to influence the work reported in this paper.
